# Activation of mitochondrial energy metabolism protects against cardiac failure

**DOI:** 10.18632/aging.100234

**Published:** 2010-11-16

**Authors:** Tim J. Schulz, Dirk Westermann, Frank Isken, Anja Voigt, Beate Laube, René Thierbach, Doreen Kuhlow, Kim Zarse, Lutz Schomburg, Andreas F. H. Pfeiffer, Carsten Tschöpe, Michael Ristow

**Affiliations:** ^1^ Department of Human Nutrition, Institute of Nutrition, University of Jena, Germany; ^2^ Department of Cardiology and Pulmology, Charité University Medicine, Campus Benjamin Franklin, Berlin, Germany; ^3^ Department of Clinical Nutrition, German Institute of Human Nutrition Potsdam-Rehbrücke, Nuthetal, Germany; ^4^ Department of Endocrinology, Diabetes and Nutrition, Charité University Medicine, Campus Benjamin Franklin, Berlin, Germany; ^5^ Institute of Experimental Endocrinology, Charité University Medicine, Campus Virchow-Klinikum, Berlin, Germany; ^6^ Current address: Joslin Diabetes Center, Harvard Medical School, Boston, MA, USA

**Keywords:** OXPHOS, cardiac failure, cardiomyopathy, insulin signaling, mitohormesis

## Abstract

Cardiac failure is the most prevalent cause of death at higher age, and is commonly associated with impaired energy homeostasis in the heart. Mitochondrial metabolism appears critical to sustain cardiac function to counteract aging. In this study, we generated mice transgenically over-expressing the mitochondrial protein frataxin, which promotes mitochondrial energy conversion by controlling iron-sulfur-cluster biogenesis and hereby mitochondrial electron flux. Hearts of transgenic mice displayed increased mitochondrial energy metabolism and induced stress defense mechanisms, while overall oxidative stress was decreased. Following standardized exposure to doxorubicin to induce experimental cardiomyopathy, cardiac function and survival was significantly improved in the transgenic mice. The insulin/IGF-1 signaling cascade is an important pathway that regulates survival following cytotoxic stress through the downstream targets protein kinase B, Akt, and glycogen synthase kinase 3. Activation of this cascade is markedly inhibited in the hearts of wild-type mice following induction of cardiomyopathy. By contrast, transgenic overexpression of frataxin rescues impaired insulin/IGF-1 signaling and provides a mechanism to explain enhanced cardiac stress resistance in transgenic mice. Taken together, these findings suggest that increased mitochondrial metabolism elicits an adaptive response due to mildly increased oxidative stress as a consequence of increased oxidative energy conversion, previously named mitohormesis. This in turn activates protective mechanisms which counteract cardiotoxic stress and promote survival in states of experimental cardiomyopathy. Thus, induction of mitochondrial metabolism may be considered part of a generally protective mechanism to prevent cardiomyopathy and cardiac failure.

## INTRODUCTION

Cardiac failure (CF) is one leading cause of death [1], and its prevalence increases with age [1]. More than seven decades ago it was proposed that the failing heart and its associated state, CF, might be caused by energy starvation [2]. Since then, a large set of published evidence has discussed the paramount importance of mitochondrial malfunction in the pathogenesis as well as late stages of cardiac failure [3,10]. In recent years, much attention has been attributed to the dysfunction of mitochondrial energy metabolism, which has not only been associated with CF but also to numerous other disorders, such as cancer, diabetes, obesity and general senescence. The mitochondrion hosts the enzymes of the Krebs cycle and the complexes of the electron transport chain (ETC) which generate ATP by oxidation of carbohydrates, fatty acids and amino acids. It therefore functions as the foremost supplier of energy substrate to maintain systemic energy balance and homeostasis.

The protein frataxin acts as an important regulator of mitochondrial energy metabolism [11] which is highly expressed in metabolically active tissues such as brain, liver, skeletal and cardiac muscle [12]. It is a nuclear encoded protein which, after N-terminal signal peptide cleavage, is located within the mitochondrial matrix. Recent studies demonstrate that frataxin is an important regulator of iron metabolism and storage within the mitochondria, and a key factor of iron-sulphur-cluster (ISC) synthesis [13]. Friedreich's Ataxia (FRDA), the most prevalent form of inherited neurodegenerative diseases, is caused by severely reduced expression of frataxin [14]. Primary symptoms are limb and gait ataxia, muscular atrophy and subsequent limb failure, carbohydrate intolerance, diabetes, and severe cardiomyopathy. Eventually, CF is fatal at an average age of 37 years [14]. A mouse model of frataxin-deficiency in the heart demonstrates a similar phenotype with cardiac hypertrophy and early adult lethality [15]. These findings are supported by the notion that cardiac dysfunction is generally associated with impaired mitochondrial energy conversion and increased formation of ROS [5,16].

We now have generated a mouse model with transgenic over-expression of the mitochondrial protein frataxin to address our hypothesis that constitutive activation of mitochondrial energy metabolism might reduce the incidence of experimental CF. We employed doxorubicin (DOX), also used as an anticancer drug in human medicine, to induce experimental cardio-myopathy. DOX is known to cause CF in humans once exceeding a cumulative dose threshold [17]. This in turn led to the use of DOX in a long-standing and standardized mouse model of cardiomyopathy, where a single injection of DOX primarily affects the myocardium and causes severe and potentially lethal CF [18,19]. We here show that transgenic activation of mitochondrial energy metabolism in the heart significantly reduces mortality in a model of DOX-induced cardiomyopathy.

## RESULTS

### Generation of a mouse model with frataxin over-expression

**Figure 1.**
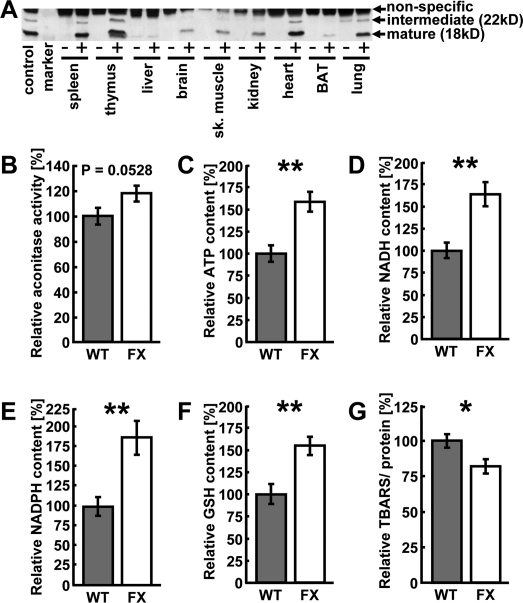
Over-expression of frataxin induces mito-chondrial metabolism and ROS defense in the heart. (**A**) Representative anti-hemagglutinin immunoblot showing several tissues from a transgene-negative littermate (“-”) and a transgenic (“+”) animal each. “Control” is a previously published cell line over-expressing frataxin [20]. (**B**) Aconitase activity measured in murine heart samples. Grey bars indicate wild-type (WT) and white bars indicate frataxin-transgenic (FX) animals (also applies to subsequent figures). (**C**) ATP, (**D**) NADH, (**E**)) NADPH, (**F**) reduced glutathione (GSH) and (**G**) thiobarbituric acid reactive substances (TBARS) contents in the hearts of wild-type and frataxin-transgenic animals. Error bars represent S.E.M., *p < 0.05, **p < 0.01, ***p < 0.001 (applies to this and all subsequent figures) (n=4).

For the purposes of this study we generated a transgenic mouse model over-expressing the mitochondrial protein frataxin. A cDNA encoding for human frataxin under the control of a ubiquitously active promoter was introduced into the murine genome to achieve a generally elevated expression of frataxin that, due to a hemagglutinin-tag, is distinguishable from expression of endogenous protein. We originally obtained 3 founder animals out of which only one was fertile and transmitted the transgene through the germ line. After confirmation of transgene presence in the genome by transgene-specific genomic PCR, we determined protein expression levels in various tissues (Figure 1A). Although a ubiquitously active promoter was used (see Methods), transgenic frataxin expression unexpectedly was restricted to a subset of tissues. Expression was found to be highest in heart, spleen, thymus and lung, where aside from the mature, processed 18 kD form of frataxin, a precursor of frataxin, sized 22 kD, could be detected through immunoblotting of the hemagglutinin-tagged transgene (Figure 1A). While brain, skeletal muscle and kidney also expressed transgenic frataxin (Figure 1A), expression was barely detectable in others, such as liver and brown adipose tissue (Figure 1A).

### Frataxin over-expression promotes cardiac mito-chondrial energy conversion

We subsequently moved on to investigate possible phenotypic changes in the heart, as this tissue usually displays the most profound aberrations in FRDA patients [14]. To investigate whether over-expression of frataxin in an animal model leads to similar changes in energy metabolism as previously reported in *in vitro* models [11,20], we determined activity of aconitase (Figure 1B), a Krebs cycle enzyme that is known to depend on ISC and hence frataxin expression. Moreover, aconitase catalyzes the initial conversion step of citric acid to isocitric acid in the Krebs cycle and therefore represents an important step of mitochondrial substrate oxidation. Animals over-expressing frataxin showed a trend towards elevated levels of aconitase activity when compared to wild-type littermates (Figure 1B) (P=0.0528).

The levels of ATP, NADH, NADPH and reduced glutathione (GSH) in hearts of wild-type and transgenic littermate animals were quantified using high performance liquid chromatography (HPLC) (Figures 1C - F). Levels of ATP, NADH and NADPH were significantly increased in animals over-expressing frataxin (Figures 1C - E) indicating an increase in mitochondrial energy conversion and mitochondrial efficiency. We next hypothesized that an apparent induction of mitochondrial energy conversion might lead to an overall increase in oxidative damage. However, the concentration of GSH, which constitutes an important fuel to drive antioxidative protection of cellular structure, was found to be increased in animals over-expressing frataxin (Figure 1F). Similarly, thiobarbituric acid reactive substances (TBARS), which represent a measure of cellular lipid peroxidation, were significantly decreased in transgenic animals (Figure 1G).

Taken together, over-expression of frataxin promotes activity of ISC-dependent aconitase activity, elevates levels of metabolic intermediates of increased energy conversion, promotes glutathione-based ROS defense and, despite this increase in mitochondrial activity, decreases markers of oxidative stress.

### Activation of mitochondrial metabolism counteracts cardiomyopathy and reduces mortality

As frataxin has been implicated in maintenance of cardiac function, we questioned whether frataxin over-expression might in turn lead to improved performance of the heart under conditions of myocardial damage, namely an application of a single dose of DOX to induce CF.

**Figure 2.**
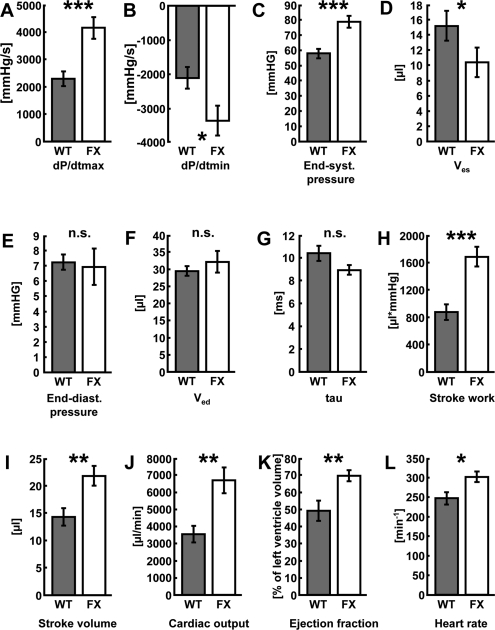
Activation of mitochondrial metabolism improves cardiac function following doxorubicin-induced cardio-myopathy. (**A**) Maximum rate of pressure development in the left ventricle (dP/dtmax), (**B**) left-ventricular maximum rate of pressure decrease (dP/dtmin), (**C**) end-systolic pressure (Pes), (**D**) end-systolic volume (Ves), (**E**) end-diastolic pressure (Ped), (**F**) end-diastolic volume (Ved), (**G**) time constant of isovolumetric left ventricular pressure decline (tau, τ). (**H**) stroke work, (**I**) stroke volume, (**J**) cardiac output, (**K**) ejection fraction, and (**L**) heart rate in wild-type vs. frataxin-transgenic animals following administration of doxorubicin. Grey bars depict wild-type litter mates, white bars frataxin-transgenic mice (n=6 each).

**Figure 3.**
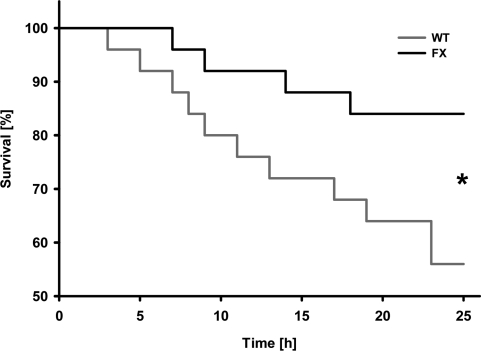
Activation of mitochondrial energy metabolism increases survival rates following doxorubicin-induced cardiomyopathy. Survival plot of animals exposed to doxorubicin. Triangles depict frataxin-transgenic (FX) animals; squares depict wild-type (WT) animals (n=24 per genotype).

We investigated whether frataxin might contribute to improved function of the heart. Hemodynamic parameters of control and transgenic hearts was assayed to monitor cardiac performance of animals either left untreated or following DOX administration. In the basal state, no differences in any of the hemodynamic parameters could be observed when comparing wild-type and transgenic animals that had not received DOX (Table 1). However, when animals were challenged with DOX prior to the measurements, transgenic animals displayed markedly improved cardiac performance. Left ventricular contraction during systole and the ventricular relaxation during diastole, end-systolic and end-diastolic pressure (Pes and Ped) as well as maximum rate of left ventricular pressure rise (dP/dtmax) and fall (dP/dtmin) indicate contractility of the ventricle. Rates of ventricular pressure rise and decline were significantly improved in the hearts of transgenic animals (Figures 2A and B). A significant improvement was also observed in Pes and end-systolic volume (Ves) (Figures 2C and D). On the other hand, no changes could be detected in the equivalent end-diastolic functions end-diastolic pressure (Ped) and end-diastolic volume (Ved) (Figures 2E and F). Tau (τ), the time constant of iso-volumetric left ventricular pressure decline was not changed significantly (Figure 2G). On the other hand, parameters of overall cardiac performance, i.e. stroke work, beat volume, cardiac output, ejection fraction and heart rate were all significantly increased in hearts with frataxin over-expression following administration of DOX (Figures 2H-L). Most importantly and in accordance with these findings, we observed a significantly decreased rate of mortality in frataxin over-expressing animals compared to wild-type littermates after DOX exposure (P=0.033) (Figure 3).

**Table 1.** Summary of hemodynamic parameters of wild-type and frataxin-transgenic animals under untreated, *i.e.* basal conditions (n=6 per group).

*Untreated animals*	Control	Transgenic	Significance
Parameter	mean (±S.E.M)	mean (±S.E.M)	P-value
**Heart rate [1/min]**	397 (±24)	395 (±26)	0.9439
**Pes [mmHg]**	96.1 (±3.8)	99.9 (±4.9)	0.5488
**Ped [mmHg]**	4.3 (±1.1)	6.1 (±1.6)	0.3603
**dP/dtmax [mmHg/min]**	7254 (±780)	7399 (±1085)	0.9137
**dP/dtmin [mmHg/min]**	-6078 (±593)	-5753 (±631)	0.7140
**Cardiac output [μl/min]**	10015 (±753)	10510 (±1354)	0.7465
**Ejection fraction [%]**	74.8 (±4.1)	70.2 (±4.4)	0.4621
**Stroke volume [μl]**	25.9 (±2.5)	26.27 (±2.2)	0.9106
**Tau [ms]**	11.9 (±0.8)	10.1 (±2.6)	0.5020
Stroke work [μl*mmHg]	2470 (±228)	2683 (±350)	0.6133
**Ves [μl]**	9.0 (±1.8)	12.0 (±2.0)	0.2878
**Ved [μl]**	34.9 (±3.5)	38.2 (±3.8)	0.5184

### Over-expression of frataxin preserves insulin signaling in DOX-induced cardiomyopathy

**Figure 4.**
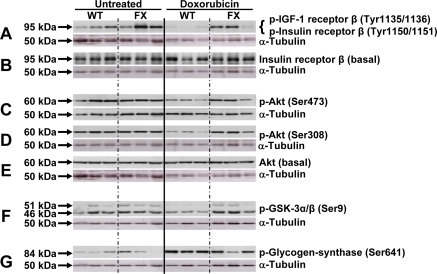
Activation of mitochondrial metabolism sustains activation of the insulin signaling cascade following doxorubicin-induced cardiomyopathy. Three animals were analyzed by western blotting for each group. Lanes 1-3: WT untreated; lanes 4-6: FX untreated; lanes 7-9: WT doxorubicin-treated; lanes 10-12: FX doxorubicin-treated. Membranes were probed with the indicated antibody, then stripped and re-probed with α-tubulin as loading control. Approximate protein band size to the left as indicated by arrows.

A close link exists between cardiac function and insulin signaling, as well as control of mitochondrial metabolism. We therefore quantified expression of components of the insulin signaling pathway in frataxin-transgenic animals before and after exposure to doxorubicin. Using immunoblotting, we analyzed expression and phosphorylation levels of IR and IGF-1R, as well as their downstream targets Akt (protein kinase B), glycogen synthase kinase-3α/β (GSK-3α/β) and glycogen synthase (GS). Expression as well phosphorylation levels of these proteins remained unchanged in frataxin-transgenic animals as compared to untreated controls in the pre-DOX state (Figure 4).

Administration of DOX to wild-type animals, however, lead to a marked reduction in phosphorylation levels of IR and IGF-1R (Figures 4A and B), as well as their downstream targets Akt and GSK-3α/β (Figures 4C to F). Phosphorylation of GSK-3α/β negatively regulates the activity of this kinase. Accordingly, phosphorylation of GS was found to be increased in wild-type animals exposed to DOX, which is known to inhibit the activity of this enzyme (Figure 4G). Meanwhile, under conditions of frataxin over-expression, we observed a reversal of the DOX-induced changes in wild-type animals. We here observed increased phosphorylation levels of the upstream insulin signaling components IR, IGF-1R and Akt (Figures 4A - E) as well as the downstream kinase GSK-3α/β (Figure 4F). According-ly, phosphorylation of GS was reduced to levels observed in the untreated hearts (Figure 4G).

Taken together, DOX-induced cardiomyopathy, cardiac function and survival was significantly improved in frataxin-transgenic mice compared to wild-type littermates. Activation of the insulin/IGF-1 signaling cascade is markedly inhibited in the hearts of wild-type mice following induction of cardiomyopathy. Importantly, transgenic overexpression of frataxin rescues the loss of insulin/IGF-1 signaling and provides a mechanism to explain enhanced cardiac stress resistance in the transgenic mice.

## DISCUSSION

We previously demonstrated that expression of frataxin correlates with mitochondrial energy metabolism *in vitro* and *in vivo*, affecting processes as diverse as longevity, diabetes, as well as growth and formation of cancer cells [11,20-23]. Therefore, we here have generated a transgenic mouse model with forced expression of frataxin. Frataxin-transgenic mice display increased mitochondrial energy metabolism in the heart, which - consistent with previously published *in vitro* data [24] - was accompanied by reduced oxidative stress. These observations indicate that over-expression of frataxin in the heart improves mitochondrial energy conversion while it reduces the amount of ROS produced during electron transport. Following the injection of DOX to induce CF we observe significantly improved cardiac function and survival in mice transgenically expressing frataxin. Moreover, we here show that activation of mitochondrial metabolism is able to sustain activation of the Akt/PKB survival pathway through the insulin/IGF-1 receptor signaling axis under conditions of ROS-induced cardiomyopathy.

**Figure 5.**
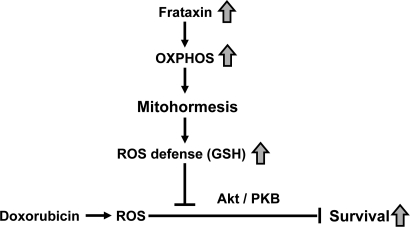
Activation of mitochondrial metabolism induces mitohormesis, hereby increasing stress resistance and survival of cardiomyopathy.

Previous findings in cells, nematodes, knock-out mice and possibly humans demonstrate that frataxin expression tightly correlates with mitochondrial activity [11,20-23,25,26]. Furthermore, we and others were able to show that increased formation of ROS can expedite health-promoting effects by inducing a hormetic response which in turn leads to improved stress resistance and/or ROS defense capacity (reviewed in [27]). In particular, this has been shown for frataxin-overexpressing cells which, similarly to the transgenic mice studied in this manuscript, exhibit reduced ROS levels and increased GSH levels despite increased mitochondrial metabolism [24]. We therefore hypothesize that frataxin acts similar to physical exercise [28] through transiently increased ROS formation by subsequently promoting mitochondrial hormesis or ‘mitohormesis' [27] (Figure 5).

It is well established that activation of insulin/IGF-1 receptor signaling exerts cardioprotective action [29-31]. Reduced insulin sensitivity and diabetes have been associated with increased incidence of CF [31] while cardiac muscle appears to respond to impaired insulin signaling by inducing mitochondrial biogenic response [32]. In the same line, treatment of rats with IGF-1 protects from cardiomyopathy induced by DOX [33], and expression of IGF-1 and its receptor is increased in rat hearts following physical exercise [34,35]. Mice carrying a deletion of both IR and IGF-1R in the skeletal muscle and heart display a severe impairment of cardiac function and die of heart failure four weeks after birth. Moreover, these animals display reduced expression levels of genes of the ETC and especially mitochondrial fatty acid oxidation, not only suggesting that insulin signaling plays an essential role on heart physiology but also is an important regulator of mitochondrial energy metabolism [36].

Activation of the Akt/PKB survival pathway has repeatedly been implicated in improved outcome following myocardial infarction. The anti-apoptotic function of Akt/PKB seems to be of particular importance to guarantee sustained heart contractility and function following reperfusion of the infarct region [37-39]. Conversely, over-expression of constitutively active Akt/PKB results in marked cardiac hypertrophy and other cardiac complications. Interestingly, and regardless of the negative growth characteristics, Akt/PKB-transgenic animals displayed significantly reduced infarct size following ischemia/reperfusion [40]. In accordance with our findings, it could be demonstrated earlier that DOX reduces Akt/PKB signaling [41,42]. Moreover, virus-mediated expression of AKT1 in mouse hearts ameliorated the effects of DOX-induced cardiomyopathy [43].

Another interesting line of evidence links reduced activity of the downstream target of Akt/PKB, GSK-3α/β, to cardioprotection. Following treatment with the polyphenolic compound resveratrol, GSK-3α/β is inhibited via phosphorylation [44]. Resveratrol protects the heart by a preconditioning mechanism acting though nitric oxide signaling [45], a process strikingly resembling the principle of hormesis. Resveratrol has been implicated in eliciting a hormetic effect in the process of ‘xenohormesis' [46].

In summary, we observe a consistent reduction of insulin/IGF-1 receptor signaling and reduced Akt/PKB phosphorylation, which has been previously reported following administration of DOX. The findings altogether indicate that over-expression of frataxin, due to its function as an activator of mitochondrial energy metabolism, rescues the DOX-induced down-regulation of insulinIGF-1 signaling. Moreover, we propose that mitochondrial metabolism, by primarily producing increased amounts of ROS, elicits a mitohormetic response which activates defense mechanisms in the heart. This effect may help to prevent cardiomyopathy and result in improved survival following treatmentwith DOX (Figure 5). Plausible future approaches for finding novel treatments of CF would therefore include the search for novel compounds that can enhance mitochondrial metabolism to induce such a mitohormetic response [4].

## MATERIALS AND METHODS

### Generation of frataxin-overexpressing animals

The cDNA encoding for the human frataxin protein was obtained and fused to a C-terminal hemagglutinin-tag as previously described [11]. The cDNA (deposited at AddGene for public access) was cloned into the NcoI and EcoRI restriction sites of the pDRIVE-CAG vector (InvivoGen, San Diego, CA, USA), which has been designed for ubiquitous expression in the mouse, hereby also replacing the original LacZ encoding sequence. The vector was then digested with the restriction enzymes SwaI and PstI to remove vector backbone and obtain the purified transgene construct for injection into the male pre-nucleus of fertilized murine oocytes derived from pure C57Bl/6 mice. All subsequent breedings were performed with C57Bl/6 wild-type animals to maintain a homogenous genetic background. Offspring was genotyped for integration of the transgene as described below. F1 offspring animals were genotyped to verify transmission through the germ line. One founder animal with stable and heritable expression of the transgene could be obtained, which was subsequently used to generate a mouse colony with stable over-expression of the frataxin protein. All animal experiments (generation of transgenes, maintenance and DOX experiments) were performed after approval by the corresponding institutional review boards.

### Animal maintenance

Animals were housed and maintained as described before [23]. For all experiments male animals at an age of 12-16 weeks were used.

### Genotyping

Breeding was designed to always yield heterozygous offspring by mating a heterozygous to a wild-type parental animal. Tail biopsies were obtained and DNA was extracted as described before [22]. PCR amplification was performed using forward and reverse primers located within the transgene with the sequences 5'-GGCTATCTTCTCCATCCAGTG-3' and 5'-TCTTATCATGTCGAGCTAGCG-3' for sense and antisense primers, respectively.

### Aconitase activity

Measurements were performed as described previously [22].

### Quantification of metabolites

Metabolites were assayed as described [47]. In brief, tissue samples were removed and snap-frozen with liquid nitrogen. Sample was homogenized in chilled acetonitrile buffer to precipitate protein. Following the removal of protein for later quantification by chloroform-extraction, the metabolite-containing fraction was subjected to HPLC separation and detection. Metabolites were identified by spiking of samples with appropriate standards. Metabolite content was normalized to protein content.

### Thiobarbituric acid reactive substances (TBARS)

Quantification was performed exactly as described previously [22].

### Doxorubicin-induced cardiomyopathy

Doxorubicin-induced cardiomyopathy was induced by intraperitoneal injection of doxorubicin (Doxo Cell, Cell Pharm, Germany, 20 mg/kg) in 24 mice with transgenic overexpression of frataxin (FX) and in 24 wild-type littermates (WT). The investigation conformed to the Guide for the Care and Use of Laboratory Animals published by the US National Institutes of Health (NIH Publication No. 85-23, revised 1985).

### Surgical procedures and hemodynamic measurements

Left ventricular (LV) function was analyzed using pressure-volume loops. The animals were anesthetized (Thiopental 125 mg/kg i.p.), intubated, and artificially ventilated. As described recently [48], a 1.4 F micro-conductance pressure catheter (Sciesence, Ontario, Canada) was positioned in the left ventricle for registration of LV pressure-volume (PV) loops in a closed-chest model. Indices of cardiac function were derived from PV data. Systolic function was quantified by LV end-systolic pressure (LVP, mmHg), dP /dt max (mmHg/s) and by ejection fraction (EF, %). Global cardiac function was quantified by the end systolic volume (ESV, μl), end diastolic volume (EDV, μl) stroke volume (SV, μl), cardiac output (CO, μl/min), and heart rate (HR, beat/min). Diastolic function was measured by LV end-diastolic pressure (LVEDP, mmHg), dP/dt min (mmHg/s), and the iso-volumetric relaxation time TAU (ms).

### Immunodetection

Detection of the various proteins by western blot was performed as described before [20]. Monoclonal antibodies were: hemagglutinin (clone 12CA5) (Roche, Mannheim, Germany), basal insulin receptor beta, Tyr1135/1136 phosphorylated IGF-1 receptor beta, and Tyr-1150/51 phosphorylated insulin receptor beta, basal glycogen synthase kinase alpha/beta, and Ser21/9 phosphorylated glycogen kinase alpha/beta (all from Cell Signaling Inc, Danvers, MA, USA). Polyclonal antibodies were: basal Akt, Ser308 phosphorylated Akt, Ser473 phosphorylated Akt, basal glycogen synthase, Ser641 phosphorylated glycogen synthase (all from Cell Signaling Inc.) and alpha-tubulin (Sigma-Aldrich GmbH, München, Germany).

Statistical analyses were performed using SPSS version 13.0. Unpaired T-tests were used to compare transgenic animals and control littermates, except for survival rates where chi-square analysis was used. P-values below 0.05 were considered statistically significant.
